# Issues to Consider Before Initiating a Project in Medical Geography

**DOI:** 10.3389/fpubh.2016.00014

**Published:** 2016-02-10

**Authors:** Jillian Hurd, Oliver Hurley, Shabnam Asghari

**Affiliations:** ^1^Primary Healthcare Research Unit, Discipline of Family Medicine, Faculty of Medicine, Memorial University of Newfoundland, St. John’s, NL, Canada

**Keywords:** errors, biases, medical geography, project design, epidemiology

This article is directed toward health professionals who have a limited background in epidemiology and geography but are interested in medical geography. Although geospatial analysis and the availability of geographic information systems is a growing field, there is little literature available regarding medical geography. Medical geography is a field that incorporates geographical and epidemiological concepts in order to investigate relationships between health and location (1). This article will be an introduction to the fundamental issues, including errors and biases, often encountered in geography and epidemiology, which inherently occur in medical geography. All issues should be considered prior to initiating a project in medical geography.

Projects in medical geography utilize spatially associated data, along with a number of different analytical techniques to investigate topics of interest. These techniques are applied in order to make assumptions about relationships or interactions within spatial data (2). Three core elements of spatial analysis are cartography, data mining, and mathematical modeling (2). Often approached in the aforementioned order, cartography is utilized first to create a map on which the results can be visualized. Once a foundation has been made, data mining attempts to reveal relationships within the data to develop a better understanding of potential outcomes that could result from the data (2). Finally, once relationships have been identified, mathematical models can be applied to the data, in order to analyze and interpret results, proposing potential answers to questions previously hypothesized (2).

Two locational data forms are used in cartography: raster data and vector data. Both can be utilized in spatial analysis; however, each format has its own advantages. When dealing with raster data, the area of space that is being investigated is divided up into a number of equally sized cells or pixels, all of which can be individually classified according to the factor(s) being investigated (e.g., temperature) (3). On the one hand, raster data represent points using a single cell and lines using a number of adjacent cells and shapes using a region of cells (4). On the other hand, vector data represent data as points, lines, and polygons (3). Points are used to represent small features, lines represent long features of small width, and polygons represent features of a given area (4). There are also two forms of attribute data used in spatial analysis: point data and regional data. Point data describe variables that are associated with a specific location, often denoted by *x* and *y* coordinates (5); whereas, regional data are associated with a defined area (5). Again, each type of attribute data has its own set of advantages and disadvantages.

Vector, raster, point, and regional data can be used individually or in combination, depending on what is being investigated and the desired outcome. In medical geography, there is a natural relationship between data points that are within a certain distance from each other. The first law of geography, defined by Waldo Tobler is “Everything is related to everything else, but near things are more related than distant things” (6). Simply put, data points that are close together are more alike than those further apart. This phenomenon occurs frequently in medical geography since we are dealing with factors that are related to space. From this, it is important to be aware of possible exposure to errors and biases throughout your project. If errors and biases are incorporated into a data set, they may promote conclusions that are inaccurate, resulting in wasted time and resources.

## Error

Errors can occur in any form of data, including locational data (location of a given observation) and attribute data (characterization of a given observation), which are both the common data types found in medical geography. Errors are primarily distinguished into two categories: sampling and non-sampling errors (7). On the one hand, sampling error occurs when the sample selected is not an accurate representation of the object under study (7). On the other hand, non-sampling error occurs when mistakes are made during the acquisition of the data, which results in an inaccurate representation of the object being studied (7). These types of errors are a lot more difficult to evaluate and are of utmost concern (8).

**Figure 1:**
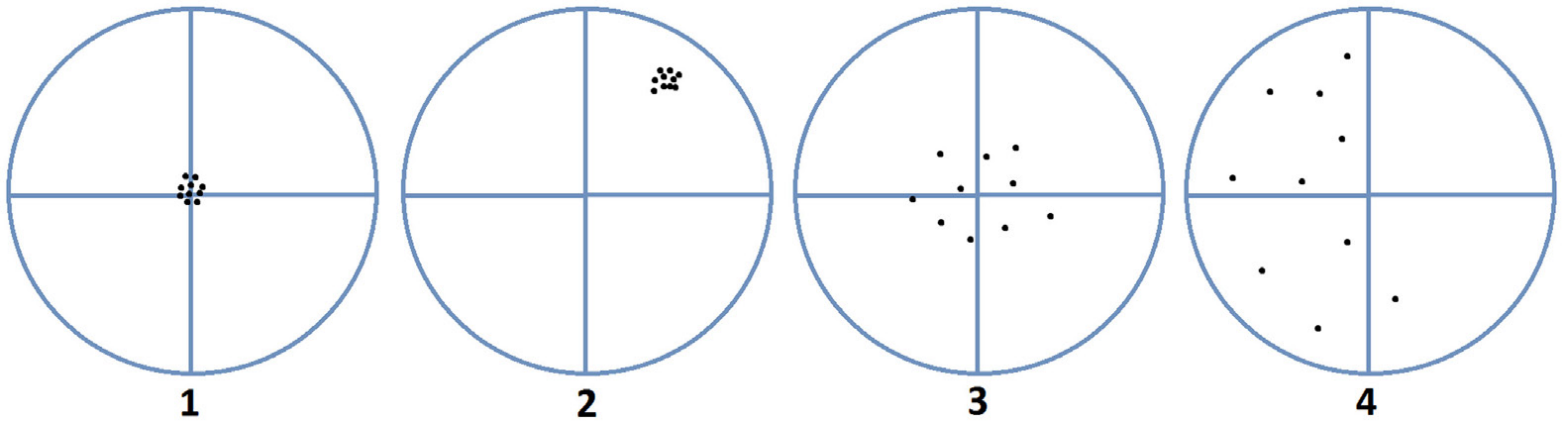
**Accuracy versus precision**. Image 1 is precise and accurate, image 2 is precise but not accurate, image 3 is accurate but imprecise, and image 4 is neither accurate nor precise (10).

Non-sampling errors are further classified into two categories: random error (unpredictable errors that result from estimation) and systematic error (reproducible errors that result from equipment or experimental design that tend to accumulate throughout the entire study) (8). Accuracy and precision are essential in minimizing systematic errors. Accuracy is defined as how close the observed value is to the true, real world value, while precision refers to how consistent the observations are (9). The diagram suggested by Pascual, 2011, seen below (Figure 1), depicts these concepts well. Any deviation from the true value being sampled can have a detrimental effect on a study’s outcome. For example, if geospatial analysis was being conducted in order to designate a quarantine area for a disease outbreak, the boundaries selected need to be accurate and precise; otherwise, some positive cases may not be contained and the disease would therefore not be properly controlled.

Systematic errors can also result from topological errors, conceptual errors (errors occurring during the transition of real world data to cartographic data), formatting errors, coverage errors (data are missed, duplicated, or falsely included), response errors (data are incorrectly requested, provided, received, or recorded), processing errors (errors during coding, capturing, editing, or inputting), estimation error, and analysis error (8).

Reducing study error should always be attempted but may not always be possible once it has already been incorporated into a study. Random error can be reduced by increasing the sample size (8). However, this technique does not resolve systematic errors that may be present in the study. Study error can also be accounted through statistical modeling techniques; however, efforts to minimize systematic error should begin at study design, before any procedures are implemented. Probability-based sample collection, as well as a conscientious study design and analysis, will help reduce the amount of systematic error in a study (10). Being aware of potential errors that could have an effect on the study results and ensuring that these are avoided will help to validate your project and reduce its overall study error.

## Bias

Bias occurs when the observed value of the data is systematically different than the true value of the data, resulting in the selection or encouragement of one sample over another (11). Bias can affect all stages of research: planning, data collection, analysis, and publication (11). In this article, we will review the most common forms of bias in spatial analysis; however, keep in mind that there are a large number of biases and their influence on results depends on the research project and study design.

### Selection Bias

During data collection, researchers can expose their study to selection bias. If there are any differences between how variables are selected or if there are any influences over what variables are selected, then the resulting sample population would be biased. Since data used in medical geography are often spatially correlated, selection bias may arise if improper sampling techniques are used. To avoid selection bias, ensure that variables are randomly selected and that there is nothing influencing the variables that are chosen.

### Information Bias

Data that are used in spatial analysis are often secondary data that were previously collected for another purpose. If these data are used for a study that do not resemble a similar objective, the data may not be a representative of the outcome of interest as original data may be (12). This form of bias can be avoided by collecting primary data specific to your study or by ensuring that the data being used are a true representation of what is being studied.

Information bias can also arise if data are missing from a data set in a non-random manner (13). An example of missing data in a non-random manner would be if people living in hard-to-reach rural areas were not included in disease status collection (13). Lacking this information could result in the assumption that people in rural areas have a lower prevalence of disease, when this is in fact, not true (13). This form of information bias can be avoided by ensuring that data are collected randomly and in even proportions across the field of study.

### Confounding Bias

Confounding bias results when an assumed association is not in fact true but instead is the result of a factor that has not been accounted for or considered (14). Cartographic confounding in medical geography can occur when the factor of interest is related to geography and a factor relating to the outcome is not randomly distributed across the study area (13). For example, if an area-level measurement is associated with an incidence of disease, however, the area-level data have missing values relating to location (i.e., rural areas are not sampled), then a relationship may be assumed, when in fact the factor of missing locational data was not taken into consideration (13). If a confounding variable has been detected, it can be accounted through statistical modeling techniques (13).

### Ecological Bias and Atomistic Fallacy

Ecological fallacy results when it is assumed that conclusions gathered from aggregated data can be equally applied to point data. For example, if regions with predominantly tall people tend to have a higher average salary, the assumption that tall people must therefore have a higher salary would be an ecological fallacy (15). Opposing this is the atomistic fallacy, where relationships found at the individual level are assumed to equally apply to the group level (16). Ecological bias and atomistic fallacy can be avoided by ensuring that conclusions are only drawn from the appropriate data and not assumed to apply to all levels of data.

### Modifiable Areal Unit Problem

Modifiable areal unit problem (MAUP) occurs when results differ based on what boundaries are selected to represent the area of interest. This could result in a biased result because the outcome within the aggregated units is subjective to the boundaries that are selected (17). There are two forms of MAUP: the scale effect and the zone effect (5). The scale effect occurs when the size of the area being analyzed changes but the data remain the same, whereas the zone effect is when the shape of the area being examined changes and therefore the data under study change as well (5). Boundaries are often predetermined as a result of constraints on the data set. From this, researchers should be aware of the boundaries that are associated with the data and reconfigure them prior to any data analysis, if necessary.

An example of MAUP would be that if a positive association between a disease and risk factor was found at a city level, but when the association was examined at the regional level, there was no longer an association (13). It is the responsibility of the researcher to determine if the difference in associations from the different boundaries is a true result or if it is due to MAUP. This can be done by ensuring that scales and zones are selected prior to data collection and analysis. Additionally, it is important to remember that the majority of factors studied in medical geography have continuous properties and therefore do not simply change once an artificial boundary is reached.

To conclude, it is important to keep in mind each form of error and bias mentioned in this article when conducting medical geography research. As previously mentioned, there is an abundance of potential errors and biases that can have an effect on research in medical geography; however, the core concepts have been mentioned here. It is the responsibility of the researchers involved to ensure that they are aware of the errors and biases mentioned in this article and that an appropriate study design is developed to minimize these problems. Ensuring the reduction of errors and biases promotes the development of accurate and concrete results, which can be defended with confidence and is a goal that all researchers should strive to achieve.

## Author Contributions

JH composed the manuscript. OH and SA provided critical ­comments on the manuscript. All authors reviewed and provided feedback on the manuscript.

## Conflict of Interest Statement

The authors declare that the research was conducted in the absence of any commercial or financial relationships that could be construed as a potential conflict of interest.
